# Patterns of Bacillary Dysentery in China, 2005–2010

**DOI:** 10.3390/ijerph13020164

**Published:** 2016-01-27

**Authors:** Han Zhang, Yali Si, Xiaofeng Wang, Peng Gong

**Affiliations:** 1 Ministry of Education Key Laboratory for Earth System Modeling, Center for Earth System Science, Institute for Global Change Studies, Tsinghua University, Beijing 100084, China; zhanghan11@mails.tsinghua.edu.cn (H.Z.); yalisi@mail.tsinghua.edu.cn (Y.S.); 2 Joint Center for Global Change Studies, Beijing 100875, China; 3 Center for Disease Surveillance and Information Services, Chinese Center for Disease Control and Prevention, Beijing 102206, China; wangxf2002abc@163.com

**Keywords:** bacillary dysentery, geographic and temporal patterns, high-risk regions, ecological drivers, seasonality, meteorological factors, urban and rural disparities, *Shigella* species

## Abstract

Although the incidence of bacillary dysentery in China has been declining progressively, a considerable disease burden still exists. Few studies have analyzed bacillary dysentery across China and knowledge gaps still exist in the aspects of geographic distribution and ecological drivers, seasonality and its association with meteorological factors, urban-rural disparity, prevalence and distribution of *Shigella* species. Here, we performed nationwide analyses to fill the above gaps. Geographically, we found that incidence increased along an east-west gradient which was inversely related to the economic conditions of China. Two large endemically high-risk regions in western China and their ecological drivers were identified for the first time. We characterized seasonality of bacillary dysentery incidence and assessed its association with meteorological factors, and saw that it exhibits north-south differences in peak duration, relative amplitude and key meteorological factors. Urban and rural incidences among China’s cities were compared, and disparity associated with urbanization level was invariant in most cities. Balanced decrease of urban and rural incidence was observed for all provinces except Hunan. *S. flexneri* and *S. sonnei* were identified as major causative species. Increasing prevalence of *S. sonnei* and geographic distribution of *Shigella* species were associated with economic status. Findings and inferences from this study draw broader pictures of bacillary dysentery in mainland China and could provide useful information for better interventions and public health planning.

## 1. Introduction

Bacillary dysentery, caused by *Shigella* bacteria, is of considerable global public health concern, especially in developing countries and low income regions [1,2]. Facilitated by the low dose of organisms required for infection [3], *Shigella* can easily be transmitted by the fecal-oral route via contaminated water, food, articles for daily use, and person-to-person contact. Poor water supply, infrastructures and sanitation, and unhygienic behaviors have been associated with dysentery [1,2,4].

China has made rapid improvements in water supply and sanitation [4], and incidence of bacillary dysentery has declined markedly, but a considerable disease burden still exists and is unevenly distributed across China [5,6]. According to the Chinese Center for Disease Control and Prevention (China CDC), bacillary dysentery is the 3rd most commonly reported infectious disease in the country, with 250,000–500,000 reported cases per year during the period 2005–2010. 

Climatic impacts on human health have drawn increasing attention recently. Seasonal variation of bacillary dysentery incidence observed around the world indicates that meteorological factors play an important role [5,7,8,9]. Meteorological factors, such as temperature, precipitation and relative humidity can affect the growth, survival and spread of the enteric bacteria in the environment [7,10,11,12,13], and meteorological factors may also affect human behaviors [14]. Temperature has been regarded as a positive predictor [9,11,12], but the reported effects of precipitation and relative humidity are inconsistent [11,15,16,17,18,19].

There are four serogroups of *Shigella* bacteria (*S. dysenteriae*, *S. flexneri*, *S. boydii* and *S. sonnei*), but their prevalence varies in time and space. *S. flexneri* is prevalent in developing countries, while *S. sonnei* dominates in the developed world [1,20]. Recently, *S. sonnei* has become more popular than *S. flexneri* in some newly industrialized regions in Thailand [2], Iran [21], South Korea [22], Taiwan [23], Vietnam [24], China [25] and Bangladesh [26]. The shift in prevalent species is considered to be associated with socioeconomic development [8].

Large numbers of bacillary dysentery infections are reported in diverse geographical areas, climate conditions and socioeconomic status in China each year. Thus, a better understanding of nationwide spatial and temporal patterns of bacillary dysentery is of great significance to ensure a better disease prevention and control. However, most published studies on cases in China merely focused on a limited spatial scope and subject matter (e.g., epidemiological or pathogenic analysis, association with environment or socioeconomic status) [7,9,25,27,28]. Only a few studies have performed nationwide estimations or analyses. Wang *et al.* [6] estimated bacillary dysentery burden in China at the provincial level (1991–2000). They found that morbidity and mortality rates were highest among the youngest and oldest age groups, and that incidence peaked in summer, with *S. flexneri* (86%) and *S. sonnei* (12%) as the major causative species. Xu *et al.* [5] assessed the geographic distribution and seasonality of bacillary dysentery in China provincially (1990–2009). They mapped changes in disease incidence over a period of 19 years and found that incidence decreased less rapidly in northwest provinces than the others. They found that relative humidity was associated with the geographic distribution of bacillary dysentery as drier provinces had greater disease burdens. They also found peak time and amplitude of incidence to differ between the north and south provinces. However, we are still not clear about the geographic distribution of bacillary dysentery incidence at the county-level and the underlying ecological drivers, as well as the association between seasonal variation of incidence and meteorological factors, whether the factors affect evenly across China. In addition, no study has yet compared dysentery burden between urban and rural China.

With regard to the prevalence of *Shigella* species, Chang *et al.* [29] conducted a systematic review and meta-analysis on causative serogroups and serotypes of *Shigella* in mainland China (2001–2010). They found that *S. flexneri* (76.2%) and *S. sonnei* (21.3%) were the major causative species and prevalence of *S. sonnei* was higher than estimated by Wang *et al.* [6] 10 years ago. They also found significantly higher rates of *S. sonnei* in east, north and northeast China than elsewhere. The variation of species proportion in time and space was attributed to economic growth and disparity of regional economic status. However, Chang *et al.* generalized their estimations to a regional level and results were not visualized.

To fill the knowledge gaps mentioned above, we conducted nationwide analyses attempting to: (1) explore the geographic distribution of bacillary dysentery incidence; (2) characterize seasonality of bacillary dysentery incidence and to assess its association with meteorological factors; (3) identify high-risk regions and their ecological drivers; (4) explore the disparities in urban and rural incidence; (5) estimate the prevalence of *Shigella* species and to analyze their spatial distribution.

## 2. Materials and Methods

### 2.1. Data Collection

Data on annual number of bacillary dysentery cases at county/district level, monthly number of bacillary dysentery cases at provincial level and corresponding population data from 2005 to 2010 were obtained from China CDC. Data on bacillary dysentery were collected by the National Notifiable Infectious Disease Reporting System (NIDR), a national surveillance system for infectious diseases established based on the Law on Prevention and Control of Infectious Disease, which includes all healthcare facilities at village, township, county, prefecture and provincial levels [30]. NIDR has provided support to academics and policy makers for decades. Meteorological data (*i.e.*, temperature, precipitation, relative humidity) were obtained from the China Meteorological Data Sharing Service System. Topographic characteristics (*i.e.*, elevation, slope) were extracted from a digital elevation model (DEM) in 90-m grids, which were downloaded from The Consultative Group for International Agricultural Research Consortium for Spatial Information (CGIAR-CSI). Digital maps of China (administrative boundaries and rivers) were downloaded from National Geometrics Center of China (NGCC). Quality of the drinking water and treatment of wastewaters and general sanitation conditions will affect incidence of shigellosis but the relevant data in detail is unavailable. It's well known that quality of drinking water and sanitation conditions has a positive correlation to economic status. In this study, we introduced an economic indicator (gross regional product) as a surrogate of these factors in our analyses. Gross regional product (GRP) of each province was acquired from National Bureau of Statistics of China, and county/district-level GRP was extracted from local statistical yearbooks.

As to prevalence of *Shigella* serogroups, no readily-made nationwide data are currently available in China [29]. In parallel with routine monitoring system NIDR, China established 20 pathogen monitoring sites for bacillary dysentery in 10 provinces [31], but these monitoring results could not fully represent China. Besides governmental pathogen monitoring reports, many published pathogen studies also contained such data. In this study, we systematically searched China National Knowledge Infrastructure (Chinese) and Web of Science (English) for papers published since January 2005, and extracted data on prevalence of *Shigella* species. “*Shigella*”, “shigellosis”, “bacillary dysentery”, “diarrhea” and “China” were used as key terms in the literature search. Included/ excluded criterion and detailed selection process are shown in Figure S1, collected data is shown in Table S1.

### 2.2. Ethical Statement

Data on bacillary dysentery used in this study were statistical data on number of infected cases provided by China CDC. The data were aggregated by administrative regions. No informed consent was required as no individual-level analysis was performed in this study. All data were anonymous and de-identified. The data were stored in a password-encrypted file in a single personal computer and authors are not authorized by the data providers to disseminate the data nor to generate copies. Thus, no approval from Institutional Review Board or equivalent ethics committee is needed.

### 2.3. Definition

Geographic demarcation of provinces in mainland China has been defined in China Health Statistics Yearbook as follows: Eastern China covers Beijing, Tianjin, Hebei, Liaoning, Shanghai, Jiangsu, Zhejiang, Fujian, Shandong, Guangdong, Hainan; Central China includes Shanxi, Jilin, Heilongjiang, Anhui, Jiangxi, Henan, Hubei, Hunan; Western China includes Sichuan, Chongqing, Guizhou, Yunnan, Tibet, Shaanxi, Gansu, Qinghai, Ningxia, Xinjiang, Guangxi, Inner Mongolia.

Definitions of urban and rural area vary in different disciplines. Limited by the basic spatial statistical unit (county/district) of the surveillance data, we adopted the administrative definition. As a typical Chinese city, urban district is urban area and rural county is rural area. There are 287 cities in China. Fourteen cities with no division of urban and rural areas were excluded. Comparison of urban and rural incidence was conducted among the remaining 273 cities. Urbanization level, the rate of urbanization of a city, is calculated as the percentage of the total population living in urban areas.

### 2.4. Methodology

#### 2.4.1. Analysis on Geographic and Temporal Patterns

A provincial incidence map was firstly drawn to have a general understanding of the distribution and change of bacillary dysentery in mainland China. Global Moran’s I was used to detect spatial autocorrelation of disease incidence. Provincial incidence and its correlation with economy (GRP) were estimated.

Seasonality of bacillary dysentery incidence in each province was characterized by analyzing the peak month, peak duration and relative amplitude. Peak duration is number of months with higher incidence than the average of the highest monthly incidence and the lowest monthly incidence in a year. Relative amplitude is the difference of the highest monthly incidence and the lowest monthly incidence divided by the average of these two incidence rates. Seasonal variation of incidence and its association with meteorological factors (temperature, precipitation and relative humidity) were estimated based on monthly provincial surveillance data. As the range of meteorological factors and dysentery incidence rate differed widely, raw data were normalized using the Feature Scaling method (Min–Max scaling) to a 0–1 range for a better exhibition. The long-term decreasing trend of bacillary dysentery incidence rate was removed by Least Square method to minimize the effect of annual variation for subsequent analysis. As temperature indices are strongly correlated [5], we only used monthly average maximum temperature as an ambient temperature indicator. Spearman correlation between incidence and each meteorological variable was primarily conducted with various lag values. The lag value with the maximum correlation coefficient was selected [16]. However, Spearman correlation coefficients of two variables can easily be affected by other variables. To measures the actual relationship between incidence and one meteorological variable, while taking away the effects of other variables, partial correlation analysis was then conducted. Partial correlation between variables *x* and *y* after adjusting for a single variable *z* is identical to that obtained from the first-order partial correlation formula:

(1) $$r_{xy.z}=\frac{r_{xy}-r_{xz}r_{zy}}{\sqrt{\left(1-r_{xz}^{2}\right)\left(1-r_{yz}^{2}\right)}}$$

where *r_xy_*, *r_xz_*, and *r_yz_* are the Spearman correlations.

The formula for higher-order partial correlations is a straightforward extension of the preceding first-order formula. Partial correlation between *x* and y controlling for both *z*_1_ and *z*_2_ can be computed as:

(2) $$r_{xy.z_{1}z_{2}}=\frac{r_{xy.z_{1}}-r_{xz_{2}.z_{1}}r_{yz_{2}.z_{1}}}{\sqrt{\left(1-r_{xz_{2}.z_{1}}^{2}\right)\left(1-r_{yz_{2}.z_{1}}^{2}\right)}}$$

where $r_{xy.z_{1}}$, $r_{xz_{2}.z_{1}}$, and $r_{yz_{2}.z_{1}}$ are first-order partial correlations.

#### 2.4.2. Identification of Endemically High-Risk Regions and Their Ecological Drivers

A detection of endemically high-risk regions in China was conducted based on county-level surveillance data. Counties with higher disease incidence than the upper quartile level of each year for no less than five years during 2005–2010 were identified as high-risk regions. Hot spot analysis (Getis-Ord General G method) was also conducted as assistance in high-risk regions identification. Presumed causal environmental factors (*i.e.*, terrain, climate and rivers) and economic status (*i.e.*, GRP) were compared between high-risk counties and low-risk counties in the same provinces to identified risk factors.

#### 2.4.3. Exploration of Disparities of Urban and Rural Incidence

We analyzed the 273 cities one by one trying to figure out how incidence changed in urban and rural areas and we classified the cities into three groups: (a) cities with consistently higher urban incidence than rural incidence; (b) cities with consistently higher rural incidence than urban incidence; (c) cities with variable trends of urban-rural disparity. Urbanization level of the cities in different groups was compared. We also compared the change of bacillary dysentery incidence in urban and rural areas on provincial level.

#### 2.4.4. Meta-Analysis on Prevalence of *Shigella* Species

Meta-analysis was executed as follows. Between-study heterogeneity was estimated by the Cochran’s Q test and I^2^ statistic. If significant heterogeneity (I^2^ > 50, *p* < 0.05) among studies existed, random effects model (DerSimonian-Laird method [32]) was utilized to estimate; otherwise, fixed effects model (Inverse Variance method [33]) was applied. For a province lacking provincial monitoring on pathogen, we merged the estimations of affiliated cities as a substitution. Prevalence of major causative *Shigella* species was estimated by cities, provinces and geographic regions. Included studies on pathogen monitoring of *Shigella* serogroups were usually conducted at a province, city or county/district level. We kept detailed notes on the study area of each study and visualized without generalizing to a provincial level or regional level.

In this section, geographic demarcation was the same as Chang *et al.* [29] in order to make a comparison: Eastern China includes Anhui, Fujian, Jiangsu, Shandong and Zhejiang; Northern China includes Beijing, Hebei, Inner Mongolia, Shanxi and Tianjin; Middle China includes Henan, Hubei, Hunan and Jiangxi; Southern China includes Guangdong, Guangxi and Hainan; Northwestern China includes Heilongjiang, Jilin and Liaoning; Southwestern China includes Chongqing, Guizhou, Sichuan, Tibet and Yunnan; Northeastern China includes Gansu, Ningxia, Qinghai, Shaanxi and Xinjiang.

Statistical Analyses were performed using IBM SPSS Statistics (Version 19.0, SPSS, Inc., Chicago, IL, USA). In the comparison analysis, data were firstly tested for normality using Shapiro-Wilk Test. If the data were normally distributed, a Two-tailed T Test was conducted. Otherwise, a non-parametric Mann-Whitney U Test was used instead. Wilcoxon Test was used for paired difference test. Bonferroni correction was performed for multiple tests and the *P*-Values were corrected. All the maps presented in this study were created with ArcGIS software (Version 10.0, ESRI Inc., Redlands, CA, USA). Some small islands that belong to China were not drawn due to lack of data. Meta-analysis was performed by the Comprehensive Meta-Analysis software (Version 3.0, Biostat, Englewood, NJ, USA). Relevant articles addressing bacillary dysentery in China were reviewed to acquire background knowledge and to explain and verify what we found. Mortality cases are not discussed as they are rare in China.

## 3. Results

### 3.1. Geographic and Temporal Patterns

**Figure 1:**
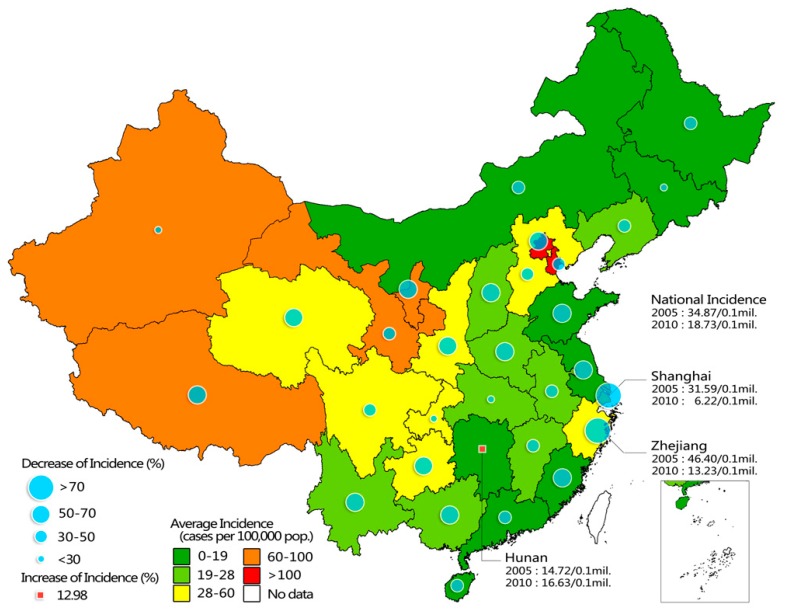
Annual average and change of bacillary dysentery incidence in each province in China, 2005–2010. Annual incidence for each year is shown in Figure S3. The map was created using the ArcGIS 10.0 software (ESRI Inc.).

National bacillary dysentery incidence decreased gradually from 34.87 cases per 100,000 residents in 2005 to 18.73 cases per 100,000 residents in 2010, but the burden was unevenly distributed geographically (Figure 1). From 2005 to 2010, provincial average incidence generally increased along an east-west gradient and was inversely associated with economy (provincial GRP) (Spearman correlation coefficient *r* = −0.471, *p* < 0.01), as provinces with higher incidence were generally concentrated in less developed western China, while incidence rates were lower in more developed eastern and central provinces (Global Moran’s Index: 0.233, *Z*-Score: 2.936, *p* < 0.01) (Figure 1 and Figure S2). However, the highest annual mean incidence was observed in Beijing (188.70 cases per 100,000 residents) and Tianjin (114.53 cases per 100,000 residents), both of which are developed regions.

During 2005–2010, incidence in each province decreased steadily, except for a slight increase in Hunan. Zhejiang and Shanghai witnessed the quickest decline of dysentery. However, Zhejiang and Shanghai fell into the yellow category due to the relatively high incidence in the early years (Figure 1 and Figure S3). The eastern China outside Beijing experienced a quicker decrease of dysentery burden than central China, with both incidence and the number of infected cases dropping rapidly to a figure lower than that of central China (Figure S2). As to prefectural-level divisions, nearly 90% (86.90%–89.32%) of *Shigella* infections were observed in China’s cities as more than 93% of Chinese inhabit in cities, and the rest Chinese live in ethnic autonomous areas. However, the incidence rate of bacillary dysentery in ethnic autonomous areas (36.50–63.79 cases per 100,000 residents), which concentrated in western China, almost doubled those of cities (17.70–33.29 cases per 100,000 residents).

**Figure 2:**
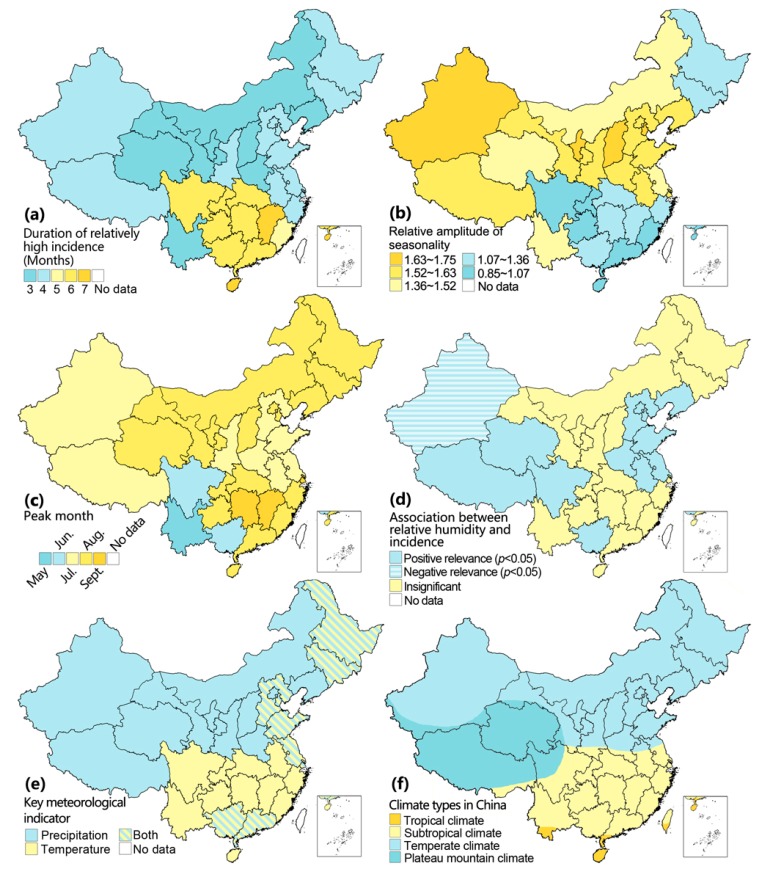
Seasonality of bacillary dysentery incidence and meteorological factors in each province: (**a**) peak duration, number of months with a higher incidence than half of peak incidence; (**b**) relative amplitude, ratio of peak and bottom incidence; (**c**) peak month; (**d**) association between relative humidity and incidence; (**e**) key meteorological indicator; (**f**) climate types in China [34]. The maps were created using the ArcGIS 10.0 software (ESRI Inc.).

Incidence rates of bacillary dysentery demonstrated obvious seasonal patterns in all provinces of mainland China, with a higher incidence in summer and autumn and lower incidence during spring and winter (Figure S4). Peak duration lasted longer with moderate amplitude in south China, while the peak duration was shorter but had greater amplitude in north China (Figure 2a,b)). The peak usually occurred in June or July, but it appeared earlier in southwest China and later in Hunan, Jiangxi and Shanghai (Figure 2c). Spearman correlation analysis indicated that either temperature or precipitation was positively related to the monthly incidence of bacillary dysentery in different province (*p* < 0.01). Relative humidity only had a significant positive correlation (*p* < 0.05) with seasonal variation of incidence in the North China Plain (Beijing, Tianjin, Hebei, Shandong and Henan) and surrounding provinces (Liaoning, Anhui, Jiangsu), Guangxi, Sichuan, and Qinghai-Tibetan Plateau, but a significant negative effect in Xinjiang (*p* < 0.01) (Figure 2d). Partial correlation coefficient between incidence and each meteorological variable is summarized in Table S2. The results indicate that temperature may play an important role in the seasonality of bacillary dysentery incidence in tropical and subtropical provinces, while precipitation may be the key meteorological indicator in temperate and plateau provinces (Figure 2e). Interestingly, in partial correlation analysis, both precipitation and temperature were significant (e.g., Tianjin, Shanghai, Guangxi, Guangdong, Hebei, Heilongjiang, Jilin, Jiangsu and Shandong) and at times nearly equivocal in some provinces (e.g., Tianjin and Shandong). Provinces of dual significance were restricted to eastern China (Figure 2e).

### 3.2. Environmental Drivers in High-Risk Regions

**Figure 3:**
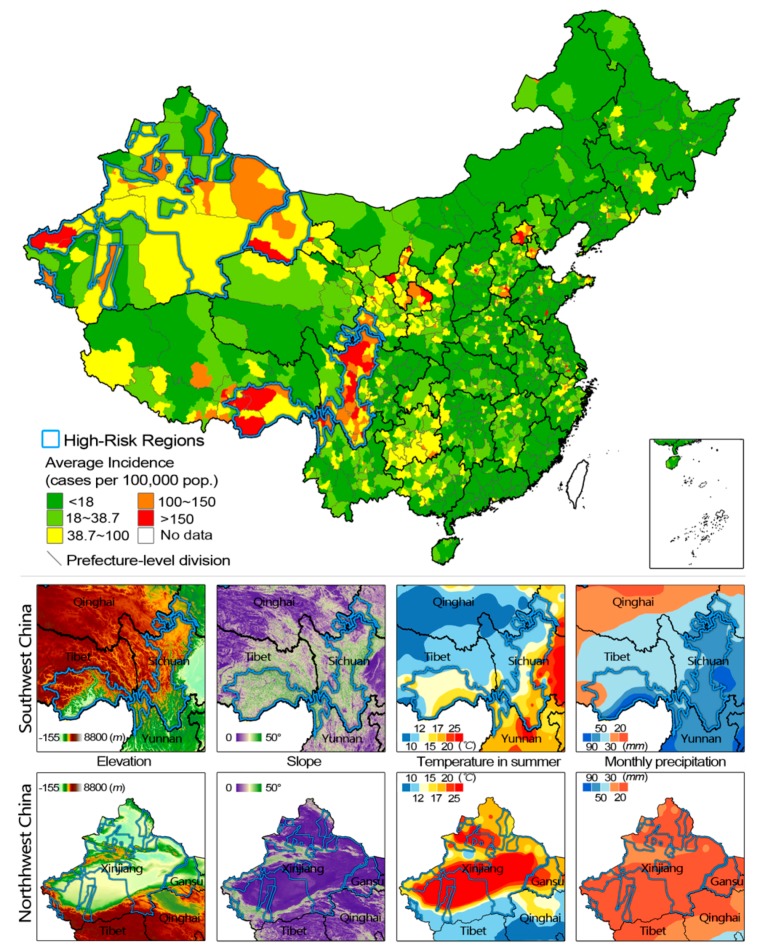
Endemically high-risk regions in southwest and northwest China and relevant environmental factors. Map was drawn at county-level and the color blocks represented annual average incidence of bacillary dysentery during 2005–2010. The two high risk regions in western China are marked in blue. Gray line: boundary of municipality cities, prefecture-level cities, autonomous prefectures, prefectures and leagues. The maps were created using the ArcGIS 10.0 software (ESRI Inc.).

Based on county/district-level surveillance data, two large high-risk regions that are consistent in time and space were identified in southwest and northwest China respectively (Figure 3). One region ran through Sichuan province from north to south, and extended to northwest Yunnan, south Gansu and southeast Tibet. The other consisted of northwest Gansu and parts of Xinjiang. Presumed relevant factors were compared between high-risk counties/districts and low-risk ones in the same provinces, and results are summarized in Table S3.

According to Table S3, high-risk regions in south Gansu, Sichuan, Yunnan and Tibet shared some similar features: (1) topographically, they were mountain areas with huge undulations and are located on the edge of the Tibetan Plateau; (2) suitable climatic conditions with warm temperature and ample precipitation; (3) backward in economic development. On the east side of the high-risk regions, cities on the lowland had more favorable climate conditions, but better economic development. Thus bacillary dysentery was not prevalent there. On the other side of the high-risk regions toward the hinterland of the Plateau, temperature and precipitation decreased in pace with the increasing of elevation, which might have restricted the reproduction and transmission of *Shigella*.

In north Gansu and Xinjiang, the terrain was flat and climate was dry. High-risk areas in north Gansu were found to be even drier and had lower drainage density than their surrounding counties with lower dysentery incidence, but other environmental conditions and county-level GRP were indistinctive. In Xinjiang, physical environment and economic status in high and low incidence regions were similar.

### 3.3. Disparity of Urban and Rural Incidence

Generally, both urban and rural incidence decreased during the period. 60.44% (165/273) of the cities had a consistently higher urban incidence than rural incidence, while 21.94% (52/273) of the cities had a consistently higher rural incidence than urban incidence. The remaining 20.51% (56/273) of the cities that had variable trends of urban-rural disparity were attributed to (a) quicker decrease of incidence in urban (21/56) or rural (7/56) areas, or continuous increase of incidence in rural areas (3/56) in some cities in Hunan province and surpassed the incidence in urban areas; (b) equivalent urban and rural incidence (12/56); (c) alternately higher incidence in urban or rural areas without regularity (13/56). Their geographic distribution is shown in Figure S5.

We found that the urban-rural disparity was related to the urbanization level of a city (Table 1). The urbanization level of the 165 cities with a higher urban incidence was significantly higher than that of the 52 cities with a higher rural incidence (*p* < 0.01).

**Table 1:** Urbanization level and urban-rural incidence disparity.

Urbanization Level	Bacillary Dysentery Incidence		
	Urban > Rural ^a^ (*n* = 165)	Urban < Rural ^b^ (*n* = 52)	Variable Trend ^c^ (*n* = 56)
Median (%) (Q1, Q3)	49.40 (40.00, 62.15)	38.95 (34.03, 48.30)	42.15 (35.28, 56.15)
Ranked Top 100 (*n* (%))	77 (46.67)	7 (13.46)	16 (28.57)
Ranked 200+ ^d^ (*n* (%))	29 (17.58)	24 (46.15)	19 (33.93)

^a^ Cities had a consistently higher urban incidence than rural incidence; ^b^ Cities had a consistently higher rural incidence than urban incidence; ^c^ Cities had variable trends of urban-rural disparity; ^d^ Urbanization level of the cities were sorted from high to low. 200+ means the urbanization level of a city ranked after the 200th among the 273 cities.

**Figure 4:**
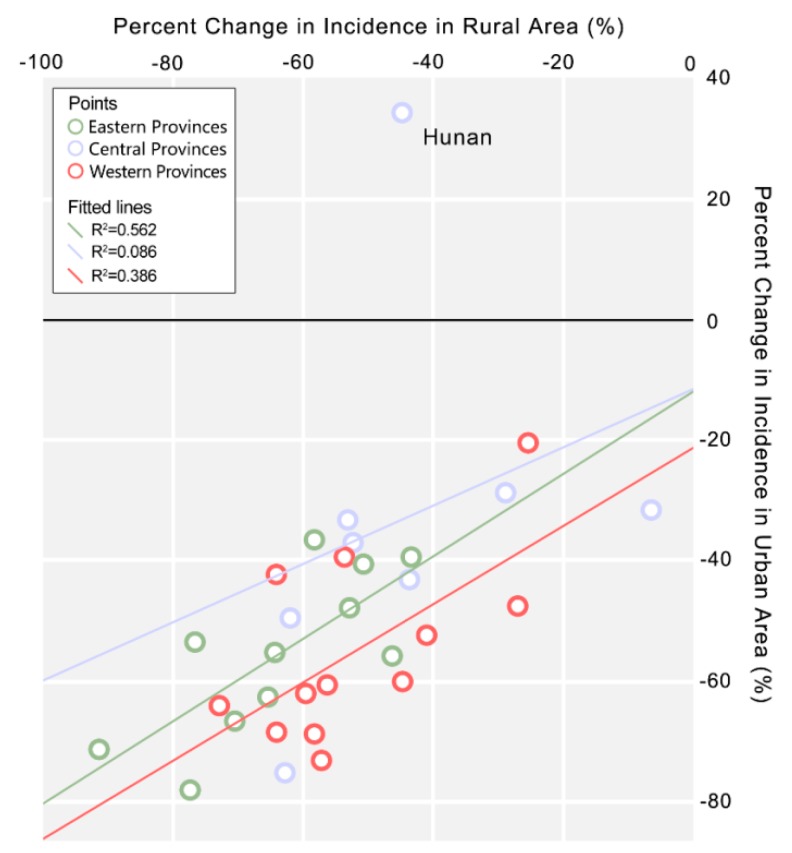
Percent change of bacillary dysentery incidence in urban and rural areas by province. Exact incidence and change of incidence in urban and rural area of each province during 2005–2010 is shown in Figure S6.

Spearman correlation analysis of the change of urban and rural incidence indicated that a balanced decrease of urban and rural incidence existed in China’s provinces (Figure 4 and Figure S6). This means that provinces with a faster decline of urban incidence usually had more rapid decrease in rural incidence (Figure 4). Balanced urban-rural improvement was better in eastern (Spearman correlation coefficient *r* = 0.75, *p* < 0.01) and western (*r* = 0.62, *p* < 0.05) China than central China (*r* = 0.29, *p* > 0.05). Detailed examination of central provinces revealed that unbalanced urban and rural improvement (urban incidence decreased while rural incidence increased gradually) happened in 7 cities of Hunan (Changsha, Chenzhou, Huaihua, Loudi, Yongzhou, Yueyang, Zhuzhou) and Changsha, Shaoyang, Yueyang witnessed rural incidence surpassing urban incidence during this period.

### 3.4. Prevalence of Shigella Species

A total of 1942 articles (1833 in Chinese, 109 in English) were included for original consideration by database searching. Evaluation on titles and abstracts selected 436 articles (397 in Chinese, 39 in English) for detailed full-text review. Finally, 220 articles (208 in Chinese, 12 in English, 263 studies in total) were included for meta-analyses. Detailed information of the included studies and relevant data are listed in Table S1.

A total of 42,355 *Shigella* isolates were analyzed. *S. flexneri* and *S. sonnei* were the two major causative species, with summarized prevalence of 71.4% (95% CI, 66.2%–77.0%) and 22.9% (95% CI, 18.6%–28.2%), respectively. *S. dysenteriae* and *S. boydii* only accounted for 1.6% (95% CI, 1.1%–2.2%) and 1.2% (95% CI, 0.9%–1.6%) of infections respectively which indicate their high rarity since 1990s [6]. Therefore, *S. dysenteriae* and *S. boydii* cases were not analyzed in this study. Estimation of the prevalence of major causative species by provinces and geographic regions were shown in Table S4.

**Figure 5:**
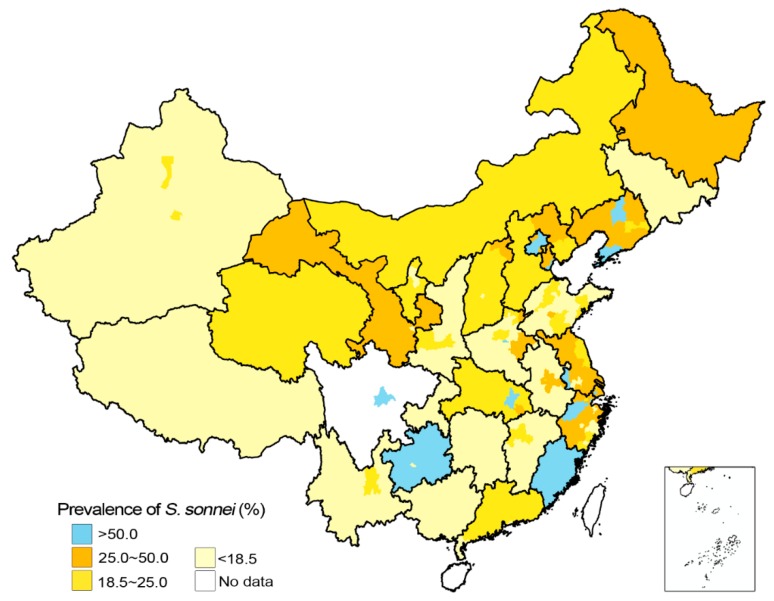
Average prevalence of *S. sonnei* in China, 2005–2010. Small color blocks are drawn according to study areas of the included studies. Background color in each province is selected according to estimation of the prevalence of *S. sonnei*. Places that are not dominated by *S. sonnei* are dominated by *S. flexneri*. The maps were created using the ArcGIS 10.0 software (ESRI Inc.).

In general, *S. sonnei* was found to be more prevalent in eastern, northern and northeastern China as compared with others regions (*p* = 0.015). Ningxia, Gansu, Qinghai in northwest China, Guizhou in southwest China and Hubei in central China were also found to have recorded relatively high rates of *S. sonnei* (Table S4). Paired difference test indicated higher rates of *S. sonnei* in many provincial capital cities and core-economic cities, such as Dalian, Harbin, Hangzhou, Hefei, Jinan, Kunming, Nanjing, Shenyang, Wuhan, Wuxi, Xi’an, as compared with the estimations of their respective provinces (*p* < 0.01). The prevalence of *S. sonnei* was mapped and is shown in Figure 5.

The overall proportion of *S. sonnei* has increased by ~10% since 1990s [6]. During 2005–2010, enormous growth of *S. sonnei* proportion was observed in Beijing (36.3% (95% CI, 30.0%–43.0%) → 80.3% (95% CI, 77.9%–82.5%)), Wuxi (35.3% (95% CI, 28.9%–42.3%) → 70.6% (95% CI, 45.8%–87.2%)) and Shanghai (31.0% (95 CI, 26.8%–35.5%) → 68.3% (95% CI, 49.9%–82.4%)). The growing prevalence of *S. sonnei* has made it more popular than *S. flexneri* and *S. sonnei* has dominated Beijing, Chengdu, Dalian, Hangzhou, Nanjing, Shanghai, Shenyang, Tianjin, Wuhan and Wuxi by 2010, all of which were among the top 15 cities by GRP in mainland China.

## 4. Discussion

The study has demonstrated that bacillary dysentery incidence increased along an east-west gradient across China. Two large endemically high-risk regions in western China and their ecological drivers were identified for the first time. Urban and rural incidences among China’s cities were compared, and disparity associated with urbanization level was invariant in most cities. Balanced decrease of urban and rural incidence was observed for all provinces except Hunan, which indicates that a better disease prevention and control in urban districts might facilitate a simultaneous improvement in adjacent rural counties. We characterized seasonality of bacillary dysentery incidence and assessed its association with meteorological factors, and it exhibited north-south differences in peak duration, relative amplitude and key meteorological factors. We conducted a systematic review and meta-analysis on causative species of *Shigella* in China, and *S. flexneri* and *S. sonnei* were found as major causative species. Increasing prevalence of *S. sonnei* and geographic distribution of *Shigella* species were associated with economic status.

Results from two large endemically high-risk regions in western China suggested the existence of longstanding risk factors. In this study, undulating terrain, appropriate climate and poor economic conditions were regarded as risk factors in southwest China. Undulating terrain is actually consistent with backward economy, as it restricts the development of regional economy. Poor economy limits construction of infrastructure, such as facilities for safe water supply, waste treatment and manure disposal, which eventually leads to deterioration of sanitation and facilitates *Shigella* transmission. Conducive climate, such as suitable temperature and ample precipitation, may support the growth, survival and spread of the bacteria in the environment. Apart from the above factors that have been explained in detail in this study, unhygienic living habits may be an additional risk factor. According to China’s Ethnic Yearbook, Tibeto-Burman ethno-linguistic groups dominate this region, and these populations have proved to be more susceptible than others as they possess many unhygienic habits such as consumption of raw food and unboiled water, and infrequent hand washing [35,36]. In northwest China, we failed to identify the risk factors using the county-level environmental data and GRP. This is probably because *Shigella* transmission is usually associated with smaller-scale environments, but the geographic extents of counties in northwestern China are much larger than elsewhere and might mask some details. According to local epidemiological investigations, the main threats in northwest China are poverty and lack of safe drinking water [37,38,39]. North Gansu has gathered over 0.15 million impoverished inner provincial migrants under organized migration plans since the 1980s. Inadequate sanitation and hygiene brings about long-term threats of bacillary dysentery to the immigrants [37]. In Xinjiang, unsafe drinking water has affected the health of millions of poor farmers and herdsmen for decades and intestinal diseases are endemic there [38,39]. There were also some small endemically high-risk countries/districts dispersed in mainland China, which were ignored in this study.

Lower dysentery incidence in rural areas than urban districts could be interpreted as improved surveillance in urban areas and underreporting due to poor NIDR in rural China [40]. However, this interpretation might not always be right. Chinese government has made great efforts to improve the availability and quality of NIDR. According to China CDC, coverage of rural NIDR improved from 66.1% in 2005 to 87% in 2011 while coverage of urban NIDR improved from 93% in 2005 to 98% in 2011. Meanwhile, omission rates of NIDR in both urban and rural health care facilities decreased gradually and were limited to around 5% by 2009 [41]. Although availability and quality of rural NIDR had improved notably, no trend on nationwide rural incidence increase and surpassing urban incidence was observed in our study except for Hunan Province. Thus, higher urban incidences in some cities were probably true and should not be simply attributed to rural underreporting and therefore ignore urban prevention and control. This inference gains more reliability when referring to developed cities with powerful rural surveillance systems, especially where rural NIDR had long achieved 100% coverage.

Globally and regionally, poor water supply, infrastructures and sanitation facilitates transmission of *Shigella* in developing countries [1,2,4] and poor rural China [42]. Our study revealed that *Shigella* infection rate was higher in less developed western China and ethnic autonomous areas than relatively more developed eastern/central China and cities respectively. However, when we focused on well-developed Chinese cities, we found that many highly urbanized cities possessed a higher incidence in urban districts where have improved water supply, infrastructure and sanitation as compared with rural counties. Similarly, Tang *et al.* found that more developed places had higher *Shigella* infection rates than elsewhere in Jiangsu, an economically advanced province in China [28]. Australians and Americans with higher income were found to be more prone to diarrhea, and incidence in urban areas was higher than rural areas [43,44]. High-income groups had increased risks of infection with *Shigella* in Denmark [45]. Findings from our study and others prompt that the main threats may be different in more advanced sub-areas or populations in areas that have already well developed.

In industrialized countries, such as the US, 69%–80% *Shigella* infections were attributed to person-to-person contact [46,47]. Densely urban populations facilitated the transmission through direct and indirect person-to-person contact, especially among young children in daycare centers and schools with poor hygienic habits and weak immunity to *Shigella* [25,43,48]. *Shigella* infection is also foodborne, and can affect the whole food supply chain including food production, processing, transport, preparation or storage [49] via unhygienic food handlers or flies. Dining out more frequently, and eating high risk food has been identified as the main risk in China and in other parts of the world [28,44]. In the US, 20%–31% *Shigella* infections are foodborne [46,47]. Recent case-control studies in urban districts in Beijing estimated that 63.42%–78.4% *Shigella* infections are attributed to ingestion of suspicious foods [50,51].

Besides of person-to-person contact and food contamination, the huge rural-urban migration, which is unique to China, might also present pressure to urban *Shigella* prevention. In previous studies, migrants were vulnerable to infectious diseases [52,53], and they had higher *Shigella* infection rate than permanent residents [27,54,55]. Urban villages, slums that have been left behind China’s fast urbanization, might be an additional threat. Urban villages provide shabby shelters for rural-to-urban migrants and local poor people, and are commonly seen in metropolises like Beijing, Shanghai, Shenzhen, Guangzhou, Wuhan, Chengdu *etc.* While China and other developing countries pay more attentions to provision of water supply and basic sanitation facilities [6,56,57,58], they should also be vigilant to “developed type” of threats, which they probably are or will be confronted with.

Two metropoli, Beijing and Shanghai have similar populations and economy, but bacillary dysentery incidence in Beijing was far higher than in Shanghai. Possible explanations for this are demographic composition, initial health status and living habits of migrants. In Shanghai, migrants’ incidence (9.9 cases per 100,000 residents in 2009) was low and slightly higher than locals’ (6.1 cases per 100,000 residents in 2009) [55] while in Beijing, incidence of migrants (127.8 cases per 100,000 residents in 2008) almost tripled that of locals (42.3 cases per 100,000 residents in 2008) [27]. According to the Sixth National Census and Statistical Yearbooks of Beijing and Shanghai, proportions of migrants in Beijing and Shanghai were equivalent (36% and 39% in 2010 respectively), but source of migration differed greatly. Migrants in Shanghai primarily came from eastern China where dysentery was not prevalent. However, more migrants (compared with that of Shanghai) move from north, northwest and northeast of China to Beijing, and dysentery was relatively more prevalent in their hometowns. Disparity of incidence between local residents in Beijing and Shanghai might be attributed to different living habits, host characteristics or prevalent *Shigella* species, and more studies are needed to arrive at a conclusion. Low underreporting rates and unsanitary catering in Beijing may also contribute [5].

In this study, seasonality of bacillary dysentery incidence was distinct in north and south China in terms of peak duration and amplitude, which is in agreement with Xu *et al.* [5]. The north-south differences implied that different climates might have taken effect. We found precipitation and temperature as the only key climatic indicators in north and south China respectively, while both were significant in eastern provinces. Inconsistently, Zhang *et al.* [9] found that temperature was the only key factor in Jinan City, the capital city of Shandong (an eastern province). This divergence is probably due to differences in scale used. The early peak time, short peak period and high amplitude in Yunnan is unique, which is probably due to the unique climatic conditions in Yunnan, and more studies are needed in this region.

The work on prevalence *Shigella* species is similar to Chang *et al.* [29], but we included more studies, and we estimated and analyzed at finer scales instead of generalizing it to a regional scale. We also visualized the estimation. Overall prevalence of *Shigella* serogroups estimated in our study generally agrees with previous estimates from the national surveillance sites in 2005 [59] and 2009 [31], and the estimate by Chang *et al.* [29]. Our estimation indicated higher rate of *S. sonnei* in east, north and northeastern China as compared with other regions, which agrees with Chang *et al.* [29]. However, Ningxia, Gansu, Qinghai in northwest China, Guizhou in southwest China and Hubei in central China were also found to possess relatively high rates of *S. sonnei*, which was not found by Chang *et al.* [29]. Increasing prevalence of *S. sonnei* and geographic distribution of *Shigella* species was found associated with regional economic status, which agrees with previous studies in China and in other parts of the world [8,29,60,61]. Prevalence of *Shigella* serogroups had turned into an industrialized model in many highly developed cities of China. However, counterexamples did exist. *S. sonnei* accounted for a fairly small proportion of bacillary dysentery in Guangzhou (19.0% (95% CI, 13.5%–25.9%)) and Shenzhen (20.0% (95% CI, 10.1%–35.8%)), core-economic cities in south China. Guizhou, one of the least developed provinces in China, is dominated by *S. sonnei* (62.4% (95% CI, 46.7%–75.9%)). The counter examples suggest that other factors may also take effect, such as local’s immunity, food habits and hygienic living conditions.

As population mobility increases and the one-child policy in China end, we cannot be too optimistic on the present decrease of *Shigella* incidence. Provision of water supply and basic sanitation facilities is important in impoverished areas, and improvement of sanitation, hygiene and food security should be strengthened in economically developed areas. Public health actions should take consideration of local climatic conditions and population characteristics.

Limitations of this research should be addressed. Generally, NIDR in both urban and rural areas have been upgraded to a good quality, but heterogeneity may exist when considering huge area and population in China. Doctor’s diagnostic capacity may also be varied and underestimation of bacillary dysentery incidence is unavoidable in passive surveillance, but the findings of this study are reasonable. Estimate on prevalence of *Shigella* species might not be fully representative of mainland China and potential biases should be recognized. Firstly, included studies were unevenly distributed in space (Table S4). Estimations might be more accurate in the places with more studies, while bias might exist where studies were insufficient. Secondly, a substituted strategy by merging prevalence of affiliated cities was applied for provinces that lacked provincial pathogen detection. Thirdly, many included studies were not population-based and some of the studies presented *Shigella* isolates fewer than 50 which may cause potential bias in sample size.

## 5. Conclusions

Geographically, bacillary dysentery incidence increased along an east-west gradient which was inversely related to the economic conditions of China. Two large endemically high-risk regions in western China and the ecological drivers were identified for the first time. Seasonality of bacillary dysentery incidence was characterized and its association with climatic factors was assessed, and it exhibited north-south differences in peak duration, amplitude and key climatic factors. Urban and rural incidences among China’s cities were compared, and the disparity was invariant in most cities and was associated with urbanization level. Balanced decrease in urban and rural incidence was observed except for Hunan Province. *S. flexneri* and *S. sonnei* were estimated as major causative species. Increasing prevalence of *S. sonnei* and geographic distribution of *Shigella* species were associated with economic status. This study draws broader pictures of bacillary dysentery in mainland China from multiple perspectives. Findings and inferences from this study could fill some knowledge gaps and provide useful information for better interventions and public health planning.

## Supplementary Materials

The following are available online at www.mdpi.com/1660-4601/13/2/164/s1. Figure S1: Search strategy to identify studies for meta-analysis; Figure S2: Bacillary dysentery incidence and number of cases in China, by geographical region, 2005–2010; Figure S3: Annual incidence of bacillary dysentery for each year, by province, 2005–2010; Figure S4: Monthly average bacillary dysentery incidence and meteorological factors by provinces, 2005–2010; Figure S5: Urban-rural disparity of bacillary dysentery incidence in China’s cities, 2005–2010; Figure S6: Bacillary dysentery incidence and change of incidence in urban and rural area of each province; Table S1: Detailed information of the included studies for meta-analysis; Table S2: Partial correlation between monthly bacillary dysentery incidence and meteorological factors; Table S3: Comparisons of environmental factors between high-risk counties and surrounding low-risk ones; Table S4: Prevalence of major causative species of shigellosis in mainland China, 2005–2010.

## Abbreviations

The following abbreviations are used in this manuscript:

CDC: Center for Disease Control and Prevention
NIDR: National Notifiable Infectious Disease Reporting System
DEM: Digital Elevation Model
CGIAR-CSI: The Consultative Group for International Agricultural Research Consortium for Spatial Information
NGCC: National Geomatics Center of China
GRP: Gross Regional Product

## References

[1] Kotloff K.L., Winickoff J.P., Ivanoff B., Clemens J.D., Swerdlow D.L., Sansonetti P.J., Sansonetti P.J., Adak G.K., Levine M.M. (1999). Global burden of *Shigella* infections: Implications for vaccine development and implementation of control strategies. Bull. World Health Organ..

[2] Von Seidlein L., Kim D.R., Ali M., Lee H., Wang X., Thiem V.D., Canh D.G., Chaicumpa W., Agtini M.D., Hossain A. (2006). A multicentre study of *Shigella* diarrhoea in six Asian countries: Disease burden, clinical manifestations, and microbiology. PLoS Med..

[3] DuPont H.L., Levine M.M., Hornick R.B., Formal S.B. (1989). Inoculum size in shigellosis and implications for expected mode of transmission. J. Infect. Dis..

[4] Carlton E.J., Liang S., McDowell J.Z., Li H., Luo W., Remais J.V. (2012). Regional disparities in the burden of disease attributable to unsafe water and poor sanitation in China. Bull. World Health Organ..

[5] Xu Z., Hu W., Zhang Y., Wang X., Tong S., Zhou M. (2014). Spatiotemporal pattern of bacillary dysentery in China from 1990 to 2009: What is the driver behind?. PLoS ONE.

[6] Wang X., Tao F., Xiao D., Lee H., Deen J., Gong J., Zhao Y., Zhou W., Li W., Shen B. (2006). Trend and disease burden of bacillary dysentery in China (1991–2000). Bull. World Health Organ..

[7] Huang D., Guan P., Guo J., Wang P., Zhou B. (2008). Investigating the effects of climate variations on bacillary dysentery incidence in northeast China using ridge regression and hierarchical cluster analysis. BMC Infect. Dis..

[8] Chompook P., Samosornsuk S., von Seidlein L., Jitsanguansuk S., Sirima N., Sudjai S., Mangjit P., Kim D.R., Wheeler J.G., Todd J. (2005). Estimating the burden of bacillary dysentery in Thailand: 36-month population-based surveillance study. Bull. World Health Organ..

[9] Zhang Y., Bi P., Hiller J.E. (2008). Weather and the transmission of bacillary dysentery in Jinan, northern China: A time-series analysis. Public Health Rep..

[10] Checkley W., Epstein L.D., Gilman R.H., Figueroa D., Cama R.I., Patz J.A., Black R.E. (2000). Effects of *EI Niño* and ambient temperature on hospital admissions for diarrhoeal diseases in Peruvian children. Lancet.

[11] Singh R.B., Hales S., Wet N., Raj R., Hearnden M., Weinstein P. (2001). The influence of climate variation and change on diarrheal disease in the Pacific Islands. Environ. Health Perspect..

[12] Patrick M.E., Christiansen L.E., Wainø M., Ethelberg S., Madsen H., Wegener H.C. (2004). Effects of climate on incidence of *Campylobacter* spp. in humans and prevalence in broiler flocks in Denmark. Appl. Environ. Microbiol..

[13] Kovats R.S., Edwards S.J., Hajat S., Armstrong B.G., Ebi K.L., Menne B. (2004). The effects of temperature on food poisoning: A time-series analysis of salmonellosis in ten European countries. Epidemiol. Infect..

[14] Hall G.V., D’Souza R.M., Kirk M.D. (2002). Foodborne disease in the New Millennium: Out of the frying pan and into the fire?. Med. J. Aust..

[15] D'Souza R.M., Becker N.G., Hall G., Moodie K.B. (2004). Does ambient temperature affect foodborne disease?. Epidemiology.

[16] Zhang Y., Bi P., Hiller J.E., Sun Y., Ryan P. (2007). Climate variations and bacillary dysentery in northern and southern cities of China. J. Infect..

[17] Li Z., Wang L., Sun W., Hou X., Yang H., Sun L., Xu S., Sun Q., Zhang J., Song H., Lin H. (2013). Identifying high-risk areas of bacillary dysentery and associated meteorological factors in Wuhan, China. Sci. Rep..

[18] Jia L., Li X., Liu G. (2007). Analysis of the association between meteorological factors and incidence of dysentery in Beijing. Modern Prev. Med..

[19] Kelly-Hope L.A., Alonso W.J., Thiem V.D., Anh D.D., Canh D.G., Lee H., Smith D.L., Miller M.A. (2007). Geographical distribution and risk factors associated with enteric diseases in Vietnam. Am. J. Trop. Med. Hyg..

[20] Gupta A., Polyak C.S., Bishop R.D., Sobel J., Mintz E.D. (2004). Laboratory-confirmed shigellosis in the United States, 1989–2002: Epidemiologic trends and patterns. Clin. Infect. Dis..

[21] Ranjbar R., Dallal M.S., Pourshafie M.R., Aslani M.M., Majdzadeh R. (2004). Serogroup distribution of *Shigella* in Tehran. Iran. J. Public Health.

[22] Seol S.Y., Kim Y.T., Jeong Y.S., Oh J.Y., Kang H.Y., Moon D.C., Kim J., Lee Y.C., Cho D.T., Lee J.C. (2006). Molecular characterization of antimicrobial resistance in *Shigella sonnei* isolates in Korea. J. Med. Microbiol..

[23] Wei H., Wang Y., Li C., Tung S.K., Chiou C. (2007). Epidemiology and evolution of genotype and antimicrobial resistance of an imported *Shigella sonnei* clone circulating in central Taiwan. Diagn. Microbiol. Infect. Dis..

[24] Vinh H., Nhu N.T., Nga T.V., Duy P.T., Campbell J.I., Hoang N.V., Boni M.F., My P.V.T., Parry C., Nga T.T.T. (2009). A changing picture of bacillary dysentery in southern Vietnam: Shifting species dominance, antimicrobial susceptibility and clinical presentation. BMC Infect. Dis..

[25] Qu M., Deng Y., Zhang X., Liu G., Huang Y., Lin C., Li J., Yan H., Li X., Jia L. (2012). Etiology of acute diarrhea due to enteropathogenic bacteria in Beijing, China. J. Infect..

[26] Das S.K., Ahmed S., Ferdous F., Farzana F.D., Chisti M.J., Leung D.T., Malek M.A., Talukder K.A., Bardhan P.K., Salam M.A. (2013). Changing emergence of *Shigella* sero-groups in Bangladesh: Observation from four different diarrheal disease hospitals. PLoS ONE.

[27] Ji G.Q., Shi J.X., Zhang W.Z., Ma Y.X., Zhang S.J. (2009). Epidemiological characteristics of bacillary dysentery in Shunyi District, Beijing from 1997 to 2008. Cap. J. Public Health.

[28] Tang F., Cheng Y., Bao C., Hu J., Liu W., Liang Q., Wu Y., Norris J., Peng Z., Yu R. (2014). Spatio-temporal trends and risk factors for *Shigella* from 2001 to 2011 in Jiangsu Province, People’s Republic of China. PLoS ONE.

[29] Chang Z., Lu S., Chen L., Jin Q., Yang J. (2012). Causative species and serotypes of bacillary dysentery in mainland China: Systematic review and meta-analysis. PLoS ONE.

[30] Yang G., Stroup D.F., Thacker S.B. (1997). National public health surveillance in China: Implications for public health in China and the United States. Biomed. Environ. Sci..

[31] Sui J., Zhang J., Sun J., Chang Z., Zhang W., Wang Z. (2010). Surveillance of bacillary dysentery in China, 2009. Dis. Surveill..

[32] DerSimonian R., Laird N. (1986). Meta-analysis in clinical trials. Control. Clin. Trials.

[33] Abrams K.R., Jones D.R., Jones D.R., Sheldon T.A., Song F. (2000). General fixed effect model—The inverse variance-weighted method. Methods for Meta-Analysis in Medical Research.

[34] Wang J.A., Zuo W. (2010). Geographic Atlas of China.

[35] Liu J., Zhang X., Wang J., Du X., Zeng J., Xu J. (2005). Contrast analysis on infectious diseases in Tibet and backland. Acta Acad. Med. Militaris Tertiae.

[36] Li K. (2007). Analysis on 1954 Bacillary dysentery cases reported in Ningnan county from 1997 to 2006. J. Prevent. Med. Inf..

[37] Li A. (2012). Analysis on the epidemic trend of legal type A and B contagious diseases in Jiuquan during 2004 to 2010. J. Med. Pest Control.

[38] Zhao H., Gu X., Zhang Q., Song X. (1998). Evaluation on the benefit of water improvement in Kashi, Xinjiang. Chin. J. Public Health.

[39] Aishan N. (2005). The existed problem and solution on safe drinking water project in rural and pastoral areas of Xinjiang. J. Hyd. Eng..

[40] Gao T., Liu G., Li X., Jia L., Liu Y., Tang Y. (2007). Analysis about epidemic situation of dysentery near upon fourteen years in Beijing. Chin. J. Prevent. Med..

[41] Liu S., Wang L., Wang X., Zhang C., Guo Q., Zhou M., Ma J. (2009). Evaluation on management and quality of communicable diseases network direct reporting in China. Dis. Surveill..

[42] Nie C., Li H., Yang L., Zhong G., Zhang L. (2014). Socio-economic factors of bacillary dysentery based on spatial correlation analysis in Guangxi Province, China. PLoS ONE.

[43] Herikstad H., Yang S., van Gilder T.J., Vugia D., Hadler J., Blake P., Deneen V., Shiferaw B., Angulo F.J. (2002). A population-based estimate of the burden of diarrhoeal illness in the United States: FoodNet, 1996–1997. Epidemiol. Infect..

[44] Hall G.V., Kirk M.D., Ashbolt R., Stafford R., Lalor K. (2006). Frequency of infectious gastrointestinal illness in Australia, 2002: Regional, seasonal and demographic variation. Epidemiol. Infect..

[45] Simonsen J., Frisch M., Ethelberg S. (2008). Socioeconomic risk factors for bacterial gastrointestinal infections. Epidemiology.

[46] Mead P.S., Slutsker L., Dietz V., McCaig L.F., Bresee J.S., Shapiro C., Griffin P.M., Tauxe R.V. (1999). Food-related illness and death in the United States. Emerg. Infect. Dis..

[47] Scallan E., Hoekstra R.M., Angulo F.J., Tauxe R.V., Widdowson M.A., Roy S.L., Jones J.L., Griffin P.M. (2011). Foodborne illness acquired in the United States—Major pathogens. Emerg. Infect. Dis..

[48] Xiao G., Xu C., Wang J., Yang D., Wang L. (2014). Spatial-temporal pattern and risk factor analysis of bacillary dysentery in the Beijing-Tianjin-Tangshan urban region of China. BMC Public Health.

[49] Bagamboula C.F., Uyttendaele M., Debevere J. (2002). Growth and survival of *Shigella sonnei* and *S. flexneri* in minimal processed vegetables packed under equilibrium modified atmosphere and stored at 7 °C and 12 °C. Food Microbiol..

[50] Zhou Y., Ma L., Xu W., Zhang H. (2007). A case-control study of bacillary dysentery in Dongcheng District Beijing City. Chin. J. Public Health.

[51] Jia L., Dou X., Wu X., Wu J., Li X., Wang Q., He X., Xu W., Sui J. (2007). Control study of risk factors of bacillary dysentery cases in Beijing. Dis. Surveill..

[52] Hu X., Cook S., Salazar M.A. (2008). Internal migration and health in China. Lancet.

[53] Gong P., Liang S., Carlton E.J., Jiang Q., Wu J., Wang L., Remais J.V. (2012). Urbanization and health in China. Lancet.

[54] Zhong X., Zhang Q. (2005). Epidemiological analysis on bacterial dysentery in Dongguan city in 2003. J. Trop. Med..

[55] Shi W., Shen H., Cui J., Xi Y., Su H., Yu L. (2011). Epidemiological analysis on bacillary dysentery during 2005 and 2009, in Minhang District, Shanghai. Shanghai J. Prevent. Med..

[56] Chompook P., Todd J., Wheeler J.G., Von Seidlein L., Clemens J., Chaicumpa W. (2006). Risk factors for shigellosis in Thailand. Int. J. Infect. Dis..

[57] Kim D.R., Ali M., Thiem V.D., Park J.K., Von Seidlein L., Clemens J. (2008). Geographic analysis of shigellosis in Vietnam. Health Place.

[58] Zhang J., Mauzerall D.L., Zhu T., Liang S., Ezzati M., Remais J.V. (2010). Environmental health in China: Progress towards clean air and safe water. Lancet.

[59] Yu H., Chang Z., Zhang L., Zhang J., Li Z., Xu J., Ran L. (2007). Analysis on the status of *Shigella* spp. Antimicrobial resistance through data from the National Shigellosis Surveillance System in China, in 2005. Chin. J. Epidemiol..

[60] Ram P.K., Crump J.A., Gupta S.K., Miller M.A., Mintz E.D. (2008). Part II. Analysis of data gaps pertaining to *Shigella* infections in low and medium human development index countries, 1984–2005. Epidemiol. Infect..

[61] Zhang W., Luo Y., Li J., Lin L., Ma Y., Hu C., Jin S., Ran L., Cui S. (2011). Wide dissemination of multidrug-resistant *Shigella* isolates in China. J. Antimicrob. Chemother..

