# Supplementary material for: Patterns of Bacillary Dysentery in China, 2005–2010
Source: Int J Environ Res Public Health. 2016 Jan 27;13(2):164. doi: 10.3390/ijerph13020164 (PMC4772184; doi:10.3390/ijerph13020164)
Supplement: Supplementary file 1 [file ijerph-13-00164-s001.pdf]

Supplementary Material: Patterns of Bacillary Dysentery in China, 2005–2010

Han Zhang, Yali Si, Xiaofeng Wang and Peng Gong

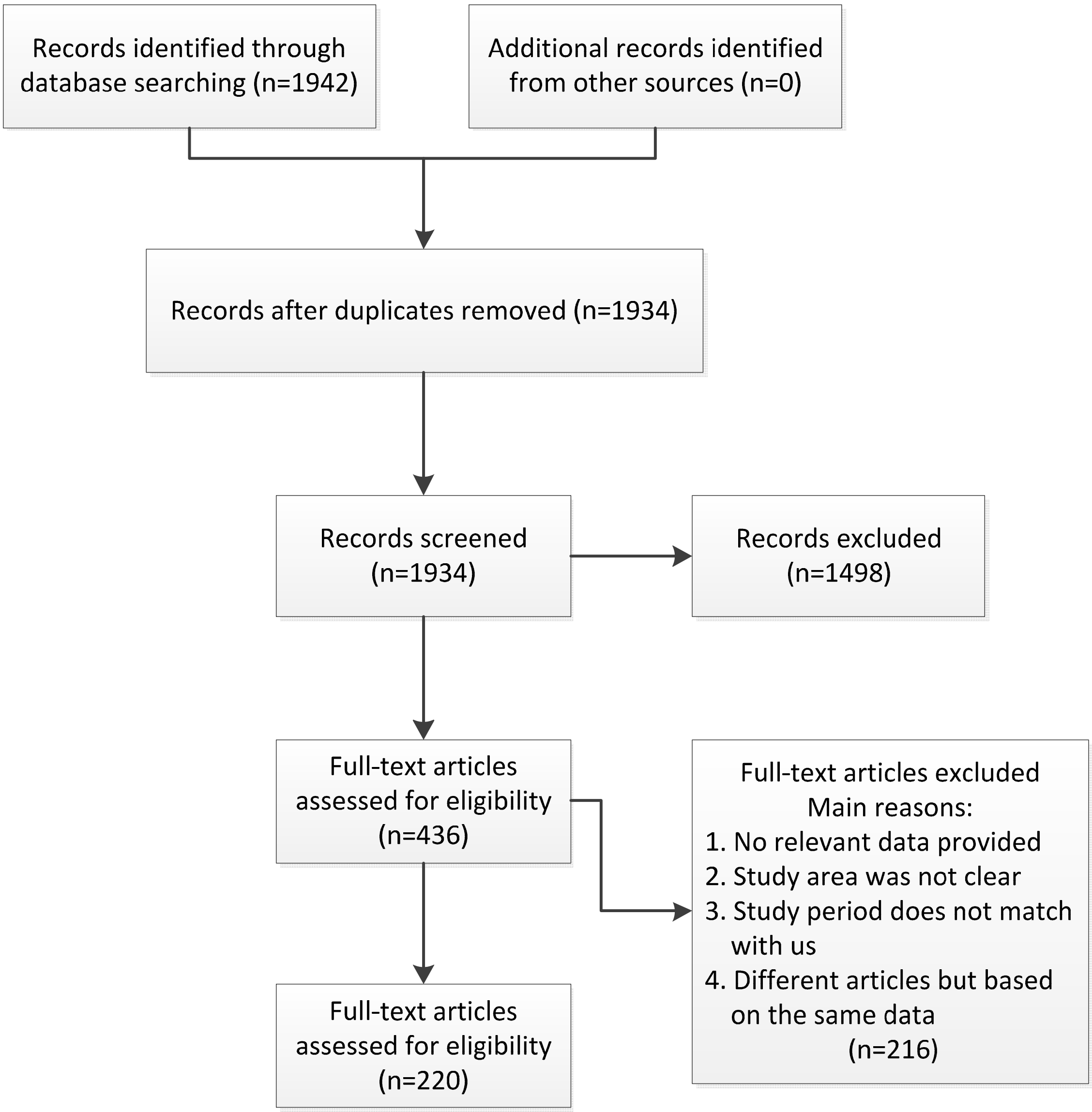

Figure S1. Search strategy to identify studies for meta-analysis.

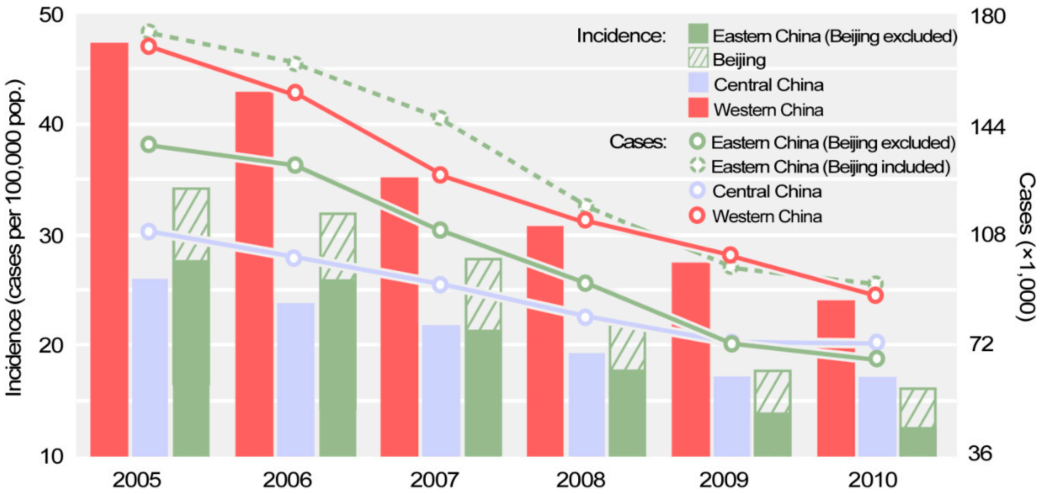

Figure S2. Bacillary dysentery incidence and number of cases in China, by geographical region, 2005–2010.

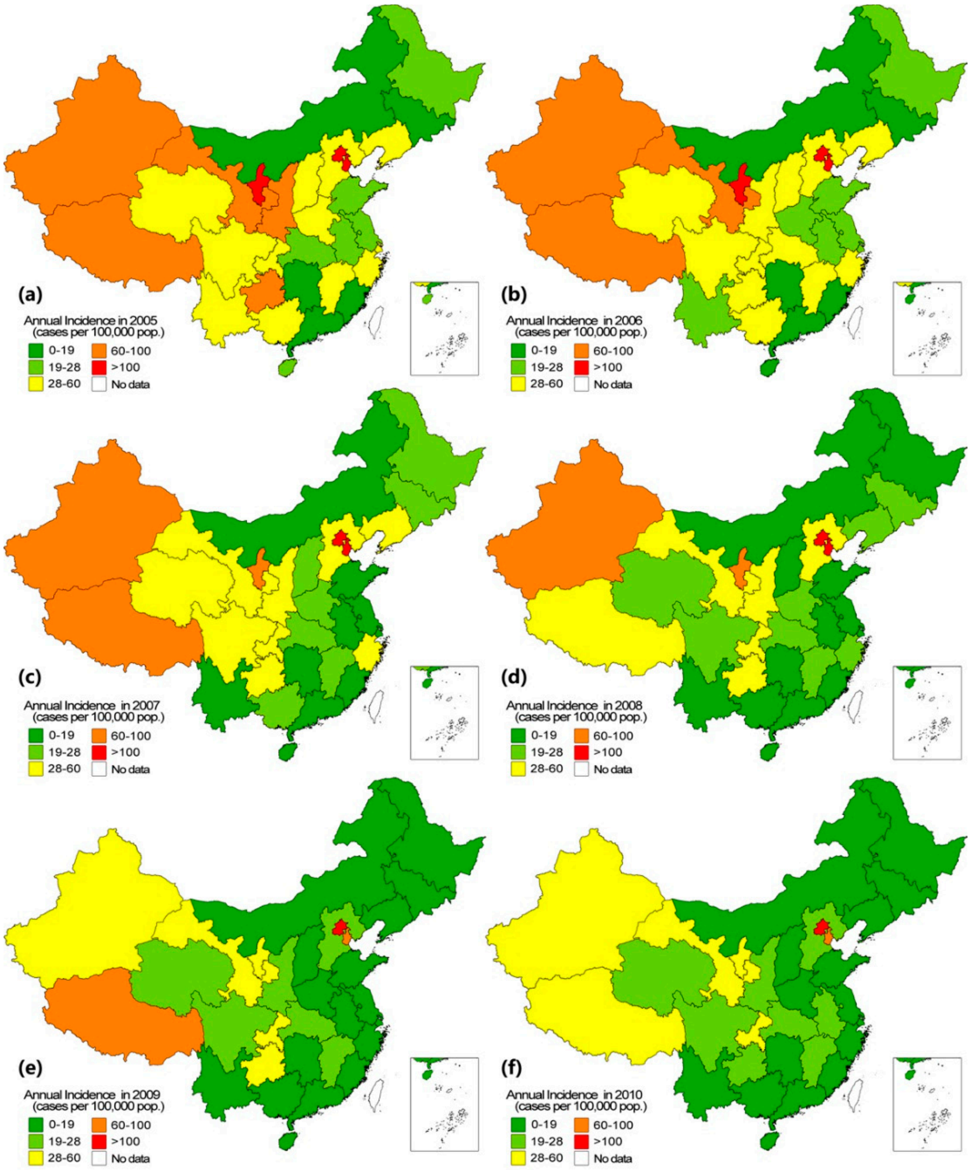

Figure S3. Annual incidence of bacillary dysentery for each year, by province, 2005–2010. The map was created using the ArcGIS 10.0 software (ESRI Inc.).

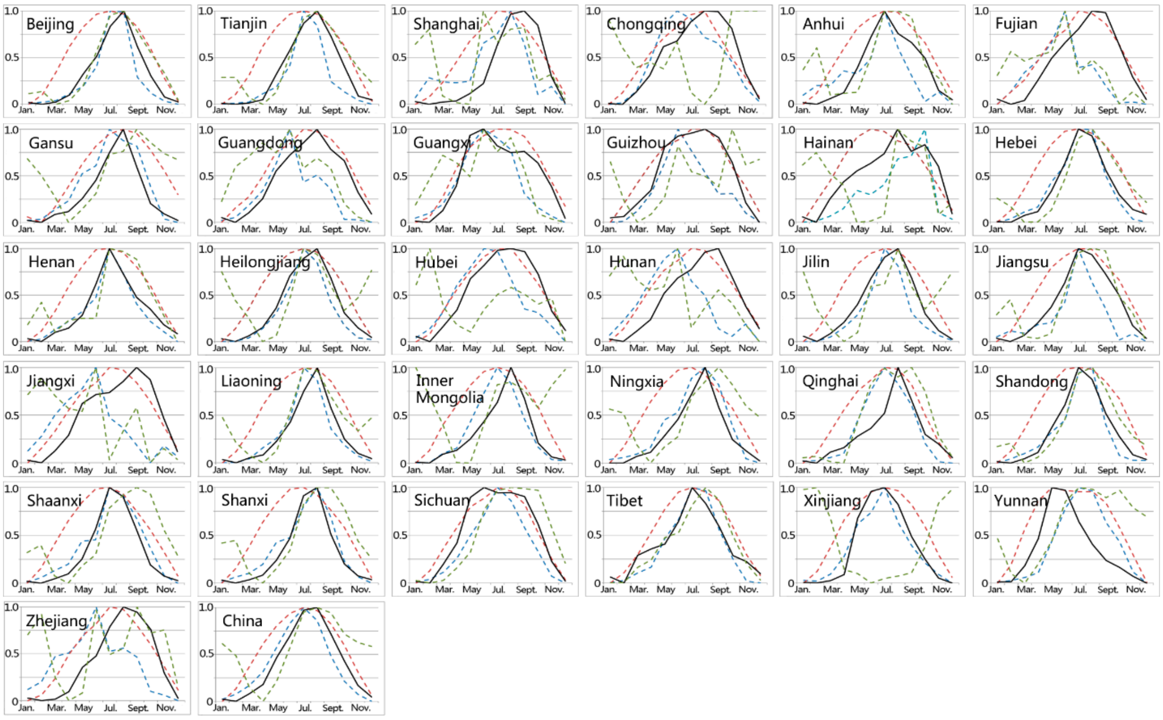

Figure S4. Monthly average bacillary dysentery incidence and meteorological factors by provinces, 2005–2010. Black solid line: Monthly bacillary dysentery incidence, Red dotted line: mean monthly temperature, Blue dotted line: mean monthly precipitation, Green dotted line: mean monthly relative humidity. Features are normalized and raw data was shown as follows (province: Incidence/ precipitation/temperature/relative humidity (minimum–maximum)) Beijing: Incidence (4.02–36.93), P (1.22–122.00), T (1.07–31.39), RH (41.89–71.96);Tianjin: Incidence (1.69–23.25), P (2.27–147.79), T (1.65–31.17), RH (47.73–74.32); Shanghai: Incidence (0.31–2.54), P (35.43–221.45), T (7.90–33.89), RH (67.92–79.31); Chongqing: Incidence (0.75–2.23), P (15.85–187.65), T (9.38–32.98), RH (74.39–83.12); Anhui: Incidence (0.48–3.70), P (26.74–260.78), T (6.51–31.64), RH (67.02–81.35); Fujian: Incidence (0.26–0.83), P (49.93–349.53), T (14.50–33.00), RH (72.36–82.68); Gansu: Incidence (1.40–13.91), P (1.67–62.56), T (0.33–27.22), RH (39.20–61.79); Guangdong: Incidence (0.43–1.07), P (26.46–260.23), T (16.77–32.15), RH (72.36–82.68); Guangxi: Incidence (0.81–3.06), P (32.42–353.89), T (15.23–33.08), RH (69.37–81.03); Guizhou: Incidence (1.65–5.24), P (21.81–225.20), T (8.93–29.48), RH (72.39–83.12); Hainan: Incidence (0.73–1.88), P (18.30–457.97), T (22.78–33.13), RH (76.21–81.32); Hebei: Incidence (0.80–6.42), P (1.66–116.18), T (−0.18–30.46), RH (46.00–73.31); Henan:Incidence (0.55–4.40), P (7.79–233.91), T (5.78–31.75), RH (58.02–79.83); Heilongjiang: Incidence (0.66–3.08), P (5.76–130.85), T (−12.78–26.86), RH (53.92–76.36); Hubei: Incidence (0.86–3.20), P (16.64–226.67), T (7.91–32.46), RH (69.13–78.43); Hunan: Incidence (0.48–2.38), P (47.16–215.83), T (8.53–33.42), RH (71.14–77.73); Jilin: Incidence (0.66–2.71), P (4.86–150.59), T (−8.08–26.90), RH (51.80–77.05); Jiangsu: Incidence (0.40–2.64), P (26.41–265.642), T (6.20–31.60), RH (66.54–80.96); Jiangxi: Incidence (0.68–3.33), P (38.22–288.47), T (10.07–34.33), RH (71.70–79.89); Liaoning: Incidence (0.64–5.26), P (4.43–177.75), T (−2.90–28.57), RH (54.28–81.78); Inner Mongolia: Incidence (0.43–2.81), P (2.12–60.65), T (−8.44–29.35), RH (36.39–58.70); Ningxia: Incidence (1.38–19.12), P (0.80–55.20), T (0.00–29.20), RH (26.18–68.66); Qinghai: Incidence (0.98–6.20), P (0.99–82.38), T (1.20–20.05), RH (37.49–58.99); Shandong: Incidence (0.34–3.29), P (3.74–196.91), T (3.61–30.18), RH (55.25–82.04); Shaanxi: Incidence (0.95–8.97), P (3.61–142.02), T (3.76–30.09), RH (52.38–77.04); Shanxi: Incidence (0.49–5.45), P (2.11–113.53), T (0.39–29.74), RH (41.99–70.73); Sichuan: Incidence (1.08–3.55), P (5.92–176.20), T (9.26–26.41), RH (57.24–73.69); Tibet: Incidence (1.38–10.61), P (1.36–84.00), T (2.80–21.33), RH (34.12–61.21); Xinjiang: Incidence (0.86–12.69), P (3.38–19.89), T (−3.72–31.43), RH (32.48–61.14); Yunnan: Incidence (0.58–4.30), P (12.60–217.60), T (18.12–27.53), RH (57.78–79.53); Zhejiang: Incidence (0.85–4.43), P (46.11–222.74), T (10.17–34.34), RH (69.40–77.94); China: Incidence (0.75–4.37), P (9.72–110.18), T (1.60–28.24), RH (48.92–63.72).

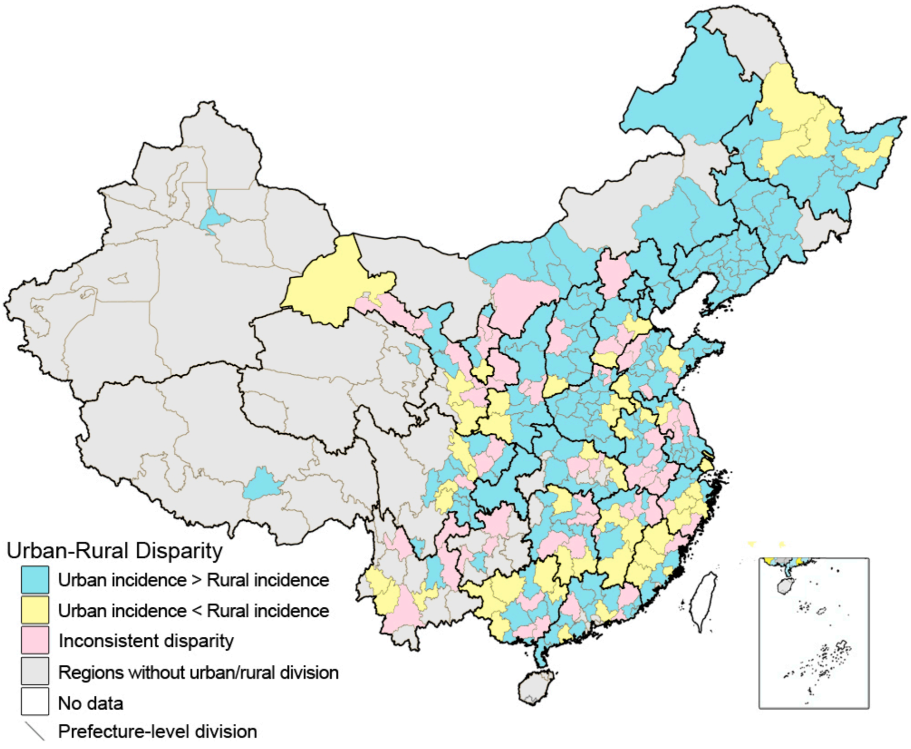

Figure S5. Urban-rural disparity of bacillary dysentery incidence in China's cities, 2005–2010. Map was draw at prefecture-level. Blue: cities had consistently higher urban incidence; Yellow: cities had consistently higher rural incidence; Pink: cities had variable trends of urban-rural disparity; Gray: ethnic autonomous areas or cities without urban/rural divisions; Gray line: boundary of cities, ethnic autonomous areas. The maps were created using the ArcGIS 10.0 software (ESRI Inc.).

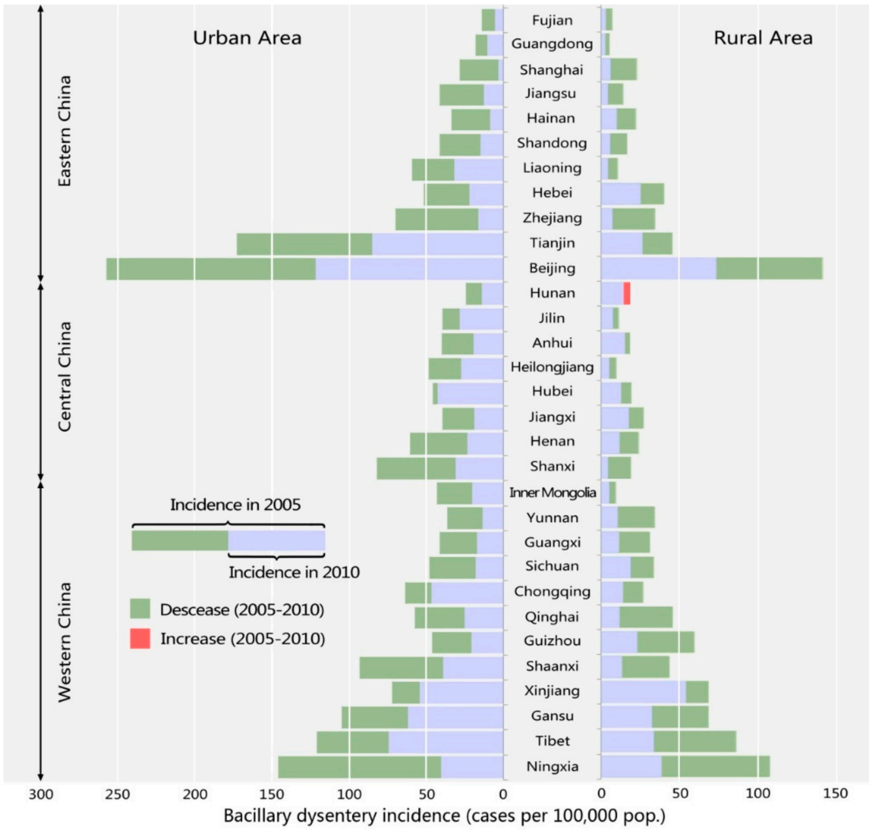

Figure S6. Bacillary dysentery incidence and change of incidence in urban and rural area of each province.

Table S2. Partial correlation between monthly bacillary dysentery incidence and meteorological factors.

Climatic Variables	Partial Correlation		
	Temperature	Precipitation	Relative Humidity
Beijing	0.550 (+1) [a]	0.820 **	0.384
Tianjin	0.712 * (+1)	0.720 *	0.555
Shanghai	0.667 * (+1)	0.776 ** (+1)	0.315
Chongqing	0.835 ** (+1)	0.142 (+1)	−0.560
Anhui	0.818 **	0.196	0.711 *
Fujian	0.808 **	0.261 (+2)	−0.320
Gansu	−0.441 (+1)	0.904 **	0.616
Guangdong	0.826 **	0.743 * (+2)	0.213
Guangxi	0.887 **	0.639 *	−0.549
Guizhou	0.858 **	0.093 (+1)	0.476
Hainan	0.820 ** (+1)	0.420	0.313
Hebei	0.740*	0.900 **	0.641 *
Henan	0.233	0.833 **	0.094
Heilongjiang	0.753* (+1)	0.900 **	0.152
Hubei	0.757 **	0.329 (+1)	0.118
Hunan	0.900 ** (+1)	0.545 (+2)	0.454
Jilin	0.712 * (+1)	0.878 *	0.151
Jiangsu	0.933 **	0.526 *	0.811 **
Jiangxi	0.766 ** (+1)	−0.102 (+3)	0.436
Liaoning	0.488 (+1)	0.785 **	0.416
Inner Mongolia	0.489 (+1)	0.712 *	0.355
Ningxia	0.206 (+1)	0.980 **	0.712 *
Qinghai	0.354 (+1)	0.763 ** (+1)	−0.592
Shaanxi	−0.118	0.820 **	−0.279
Shandong	0.801 **	0.876 **	0.428
Shanxi	0.008 (+1)	0.880 **	0.191
Sichuan	0.781 **	−0.065	0.228
Tibet	0.298	0.738 *	−0.016
Xinjiang	0.374	0.828 **	0.364
Yunnan	0.694 *	0.022	−0.074
Zhejiang	0.779 ** (+1)	−0.084 (+2)	0.166

** Significant at 0.01 level; * Significant at 0.05 level; [a] (+n) means *n* month (s) prior.

Table S3. Comparisons of environmental factors between high-risk counties and surrounding low-risk ones.

Environmental Factors	High-Risk Counties (95% CI)	Low-Risk Counties (95% CI)	*p*-Value [a]
**Sichuan** (compared with eastern Sichuan) [b]	***n* = 47** [e]	***n* = 125**	
Mean annual temperature (°C)	12.05 (10.72, 13.37)	16.87 (16.62, 17.13)	0.000 **
Summer average temperature (°C)	16.59 (15.45, 17.73)	25.49 (25.04, 25.93)	0.000 **
Monthly average precipitation (mm)	68.71 (64.65, 72.77)	84.02 (81.32, 86.73)	0.000 **
Mean annual relative humidity (%)	63.96 (61.64, 66.28)	76.04 (75.37, 76.71)	0.000 **
Elevation (mean) (m)	1652.75 (1435.92, 1869.58)	482.29 (422.60, 541.98)	0.000 **
Elevation (s.d.) [c]	1361.36 (1209.44, 1513.28)	307.69 (252.64, 362.73)	0.000 **
Slope (degree)	22.80 (21.37, 24.23)	9.87 (8.64, 11.10)	0.000 **
Drainage density (m/km^2) [d]	46.04 (37.32, 54. 77)	53.61 (47.01, 60.21)	0.152
GRP2010 (billion Yuan)	3.36 (1.91, 4.81)	12.32 (10.46, 14.18)	0.000 **
**Sichuan** (compared with western Sichuan)	***n* = 47**	***n* = 9**	
Mean annual temperature (°C)	12.05 (10.72, 13.37)	5.79 (2.96, 8.61)	0.001 **
Summer average temperature (°C)	16.59 (15.45, 17.73)	10.80 (9.42, 12.18)	0.001 **
Monthly average precipitation (mm)	68.71 (64.65, 72.77)	50.35 (46.35, 54.36)	0.000 **
Mean annual relative humidity (%)	63.96 (61.64, 66.28)	54.51 (50.65, 58.37)	0.003 **
Elevation (mean) (m)	1652.75 (1435.92, 1869.58)	2161.39 (1680.95, 2641.82)	0.066
Elevation (s.d.)	1361.36 (1209.44, 1513.28)	2082.97 (2018.41, 2147.53)	0.000 **
Slope (degree)	22.80 (21.37, 24.23)	19.92 (17.33, 22.51)	0.107
Drainage density (m/km^2)	46.04 (37.32, 54. 77)	48.62 (36.21, 61.02)	0.830
GRP2010 (billion Yuan)	3.36 (1.91, 4.81)	0.45 (0.35, 0.55)	0.001 **
**Tibet**	***n* = 17**	***n* = 48**	
Mean annual temperature (°C)	8.65 (7.72, 9.59)	5.37 (4.64, 6.10)	0.000 **
Summer average temperature (°C)	13.38 (10.81, 15.96)	10.27 (9.62, 10.93)	0.010 **
Monthly average precipitation (mm)	41.55 (36.22, 46.88)	36.67 (34.10, 39.24)	0.101
Mean annual relative humidity (%)	51.35 (46.62, 56.08)	46.35 (44.15, 48.55)	0.049 *
Elevation (mean) (m)	4184.45 (3783.94, 4584.97)	4744.59 (4667.25, 4821.93)	0.000 **
Elevation (s.d.)	700.52 (529.22, 871.82)	447.28 (412.65, 481.91)	0.000 **
Slope (degree)	23.10 (21.10, 25.11)	17.72 (16.30, 19.13)	0.000 **
Drainage density (m/km^2)	24.33 (17.58, 31.08)	29.10 (26.26, 31.95)	0.121
GRP2010 (billion Yuan)	0.62 (0.26, 0.99)	0.59 (0.40, 0.78)	0.464
**Yunnan**	***n* = 7**	***n* = 122**	
Mean annual temperature (°C)	13.12 (9.68, 16.55)	17.44 (17.00, 17.88)	0.000 **
Summer average temperature (°C)	19.86 (17.10, 22.62)	22.44 (22.09, 22.79)	0.039 *
Monthly average precipitation (mm)	82.73 (57.51, 107.95)	84.59 (80.70, 88.49)	0.975
Mean annual relative humidity (%)	67.59 (62.94, 72.24)	70.36 (69.48, 71.29)	0.169
Elevation (mean) (m)	2999.76 (2575.22, 3424.29)	1747.99 (1673.75, 1822.24)	0.000 **
Elevation (s.d.)	661.26 (599.84, 722.69)	338.98 (316.16, 361.80)	0.000 **
Slope (degree)	27.28 (24.04, 30.52)	15.91 (15.16, 16.67)	0.000 **
Drainage density (m/km^2)	44.97 (35.36, 54.59)	38.88 (34.76, 43.01)	0.377
GRP2010 (billion Yuan)	1.86 (0.46, 3.26)	5.93 (4.43, 7.43)	0.009 **
**Gansu** (south)	***n* = 8**	***n* = 19**	
Mean annual temperature (°C)	5.24 (2.09, 8.39)	9.15 (7.68, 10.62)	0.002 **
Summer average temperature (°C)	15.60 (12.40, 18.79)	20.28 (18.97, 21.58)	0.002 **
Monthly average precipitation (mm)	46.67 (42.86, 50.49)	43.23 (41.36, 45.11)	0.057
Mean annual relative humidity (%)	63.26 (61.08, 65.45)	65.02 (63.36, 66.67)	0.056
Elevation (mean) (meter)	2163.02 (1481.73, 2844.30)	1315.92 (1087.88, 1543.97)	0.002 **
Elevation (s.d.)	1241.12 (705.90, 1776.34)	998.55 (837.48, 1159.62)	0.044 **
Slope (degree)	18.22 (12.90, 23.53)	15.92 (13.36, 18.50)	0.345
Drainage density (m/km^2)	24.93 (17.52, 32.33)	31.26 (22.56, 39.96)	0.030 *
GRP2010 (billion Yuan)	0.83 (0.55, 1.12)	1.97 (1.36, 2.57)	0.006 **

Table S3. *Cont.*

**Environmental Factors**	**High-Risk Counties (95% CI)**	**Low-Risk Counties (95% CI)**	***p*-Value** [a]
**Gansu** (north)	***n*** **= 5**	***n*** **= 9**	
Mean annual temperature (°C)	7.72 (5.92, 9.51)	7.36 (5.90, 8.82)	0.739
Summer average temperature (°C)	22.86 (20.03, 25.69)	21.83 (19.82, 23.83)	0.472
Monthly average precipitation (mm)	5.46 (3.57, 7.35)	14.60 (9.32, 19.88)	0.006 **
Mean annual relative humidity (%)	40.81 (36.01, 45.61)	49.72 (47.91, 51.50)	0.004 **
Elevation (mean) (m)	1265.12 (707.86, 1822.39)	1418.35 (784.37, 2052.33)	0.947
Elevation (s.d.)	990.99 (381.02, 1600.96)	584.35 (285.15, 883.55)	0.162
Slope (degree)	3.77 (0.80, 6.75)	4.88 (0.77, 8.98)	0.841
Drainage density (m/km^2)	14.64 (4.55, 24.72)	28.07 (21.38, 34.76)	0.014 *
GRP2010 (billion Yuan)	4.56 (0.67, 9.57)	4.71 (1.69, 7.74)	0.947
**Xinjiang**	***n*** **= 48**	***n*** **= 50**	
Mean annual temperature (°C)	8.83 (7.92, 9.74)	9.28 (8.44, 10.12)	0.593
Summer average temperature (°C)	23.71 (22.70, 24.73)	24.33 (23.80, 24.86)	0.826
Monthly average precipitation (mm)	13.57 (11.03, 16.10)	11.95 (9.88, 14.02)	0.062
Mean annual relative humidity (%)	51.53 (49.27, 53.79)	51.27 (49.11, 53.42)	0.895
Elevation (mean) (m)	1530.83 (1288.02, 1773.63)	1532.71 (1279.41, 1786.02)	0.541
Elevation (s.d.)	638.17 (521.66, 754.68)	601.36 (456.91, 745.81)	0.893
Slope (degree)	7.58 (5.88, 9.28)	6.09 (4.68, 7.49)	0.241
Drainage density (m/km^2)	22.99 (17.77, 28.21)	24.73 (18.12, 31.34)	0.876
GRP2010 (billion Yuan)	4.62 (3.49, 5.76)	5.63 (3.20, 8.05)	0.524

** Significant at 0.01 level; * Significant at 0.05 level; [a] Data were tested for normality using Shapiro-Wilk Test. If the data were approximately normally distributed, a Two-tailed T Test was conducted to compare the environmental variables between high incidence areas and surrounding low incidence areas. Otherwise, a non-parametric Mann-Whitney U Test was used instead. Bonferroni correction had been performed for multiple tests of statistical significance on the same data in Sichuan prov ince, and the *P*-Values had been corrected; [b] High-risk regions in Sichuan located in the middle part of Sichuan, while eastern and western parts of Sichuan are low-risk regions. As climatic factors, geographical factors, social factors are quite different between eastern and western Sichuan, we compared environmental variables between the middle high-risk regions and low-risk regions in eastern and western Sichuan separately; [c] Standard deviation of elevation of each spatial unit, which presents the topographic fluctuation; [d] Total length of rivers per unit area; [e] n, number of counties or districts involved in comparison.

Table S4. Prevalence of major causative species of shigellosis in mainland China, 2005–2010.

	No. of Studies	No. of Isolates	*S. flexneri*				*S. sonnei*			
			Prevalence (95 CI) (%)	*n* [a]	Heterogeneity Test		Prevalence (95 CI) (%)	*n*	Heterogeneity Test	
					I^2 (%)	*p*			I^2 (%)	*p*
**Stratified by Province**										
Beijing	42	8328	39.7% (35.4%–44.1%)	3704	92.1	0	58.8% (54.1%–63.2%)	4528	92.5	0
Tianjin	4	231	52.0% (45.5%–58.4%)	120	31.0	0.23	47.1% (40.7%–53.6%)	109	25.6	0.26
Shanghai	27	9409	61.5% (56.8%–66.1%)	5406	94.8	0	38.4% (33.9%–43.2%)	4002	94.8	0
Chongqing	1	119	87.4% (80.1%–92.3%)	104	0	1.00	12.6% (7.7%–19.9%)	15	0	1.00
Anhui	12	1323	84.3% (75.1%–90.6%)	1142	90.6	0	14.6% (8.6%–23.6%)	167	90.5	0
Fujian	12	428	37.5% (33.0%–42.3%)	160	2.8	0.42	62.5% (57.7%–67.0%)	268	2.8	0.42
Gansu	12	3181	64.3% (56.8%–71.2%)	1930	92.4	0	33.4% (26.4%–41.3%)	1177	93.3	0
Guangdong	6	512	77.4% (67.7%–84.8%)	389	73.1	0	17.6% (10.6%–27.9%)	110	77.7	0
Guangxi	1	117	86.3% (78.8%–91.5%)	101	0	1.00	13.7% (8.5%–21.2%)	16	0	1.00
Guizhou	11	519	36.8% (23.2%–53.0%)	200	85.1	0	62.4% (46.7%–75.9%)	317	84.6	0
Hebei	5	403	74.6% (70.0%–78.6%)	302	36.7	0.18	22.6% (18.7%–27.0%)	90	0	0.44
Heilongjiang	24	4020	70.6% (62.3%–77.7%)	3298	90.8	0	26.9% (20.2%–35.0%)	685	86.5	0
Henan	4	142	81.8% (76.5%–86.1%)	102	54.0	0.09	17.3% (13.7%–21.5%)	37	3.34	0.38
Hubei	1	37	78.4% (62.4%–88.8%)	29	0	1.00	21.6% (11.2%–37.6%)	8	91.4	0
Hunan	1	120	85.0% (77.4%–90.3%)	102	0	1.00	10.8% (6.4%–17.8%)	13	0	1.00
Inner Mongolia	2	125	81.6% (73.8%–87.5%)	102	0	0.85	18.4% (12.5%–26.2%)	23	0	0.85
Jiangsu	17	4997	69.9% (65.0%–74.5%)	3565	89.7	0	28.8% (24.3%–33.8%)	1405	90.0	0
Jiangxi	2	277	75.2% (30.2%–95.5%)	188	97.0	0	16.8% (7.2%–34.5%)	52	86.9	0
Jilin	2	114	74.2% (32.8%–94.5%)	84	93.1	0	11.4% (6.8%–18.7%)	13	0	0.78
Liaoning	3	134	55.1% (35.8%–72.9%)	75	77.6	0.01	41.7% (20.1%–67.1%)	55	86.2	0
Ningxia	1	89	76.4% (66.5%–84.1%)	68	0	1.00	23.6% (15.9%–33.5%)	21	0	1.00
Qinghai	5	319	79.5% (74.7%–83.7%)	255	17.2	0.31	20.5% (16.3%–25.3%)	64	17.2	0.31
Shaanxi	8	1118	76.3% (61.5%–86.6%)	922	91.0	0	13.3% (5.4%–29.2%)	122	93.5	0
Shandong	11	2086	82.3% (76.6%–86.8%)	1612	89.0	0	15.7% (12.1%–20.2%)	400	83.8	0
Shanxi	8	431	81.1% (73.9%–86.6%)	346	51.4	0.04	18.9% (13.4%–26.1%)	85	51.4	0.05
Tibet	2	50	97.9% (86.4%–99.7%)	50	0	0.65	2.1% (0.3%–13.6%)	0	0	0.65
Xinjiang	8	1315	86.6% (77.2%–92.5%)	1188	89.7	0	9.2% (5.2%–15.8%)	98	85.3	0
Yunnan	2	246	96.3% (25.9%–99.9%)	225	89.5	0	3.5% (0.1%–70.5%)	19	88.8	0
Zhejiang	26	2111	63.0% (55.6%–69.9%)	1254	89.4	0	34.9% (28.2%–42.4%)	824	89.5	0

Table S4. *Cont.*

	No. of Studies	No. of Isolates	*S. flexneri*				*S. sonnei*			
			Prevalence (95 CI) (%)	*n* [a]	Heterogeneity Test		Prevalence (95 CI) (%)	*n*	Heterogeneity Test	
					I^2 (%)	*p*			I^2 (%)	*p*
**Stratified by Geographic Area**										
East	105	20354	64.5% (53.5%–77.9%)	13139	96.7	0	29.5% (19.7%–44.3%)	7066	97.5	0
North	61	9518	63.5% (50.1%–80.6%)	4574	97.4	0	30.0% (19.0%–47.2%)	4835	97.3	0
Central	28	4454	82.6% (79.0%–86.5%)	3617	0	0.78	16.3% (12.9%–20.6%)	758	15.0	0.32
South	7	629	82.4% (74.2%–91.6%)	490	60.2	0.11	15.4% (11.1%–21.4%)	126	0	0.45
Northwest	34	6022	76.7% (69.9%–84.1%)	4363	72.2	0.01	19.7% (13.4%–29.0%)	1482	83.1	0
Southwest	16	934	80.9% (66.2%–98.8%)	579	83.7	0	13.1% (3.2%–53.4%)	351	93.5	0
Northeast	9	390	69.3% (62.5%–76.8%)	261	0	0.41	23.3% (12.2%–44.2%)	105	83.4	0
**Total**										
China	260	42301	71.4% (66.2%–77.0%)	27023	94.2	0	22.9% (18.6%–28.2%)	14723	95.8	0

[a] *n*, number of isolates of *S. flexneri* or *S. sonnei*; In order to reduce the bias caused by uneven distribution of included studies, we firstly estimated prevalence of major causative species in each province. Estimation of geographic areas and mainland China was based on estimation of provinces. Two studies in Wuhan and one study in Chengdu was excluded in stratified analysis, as which could cause obvious bias on estimation of Hubei and Sichuan Province. No study in Sichuan and Hainan Province was included in stratified analysis.

© 2016 by the authors; licensee MDPI, Basel, Switzerland. This article is an open access article distributed under the terms and conditions of the Creative Commons by Attribution (CC-BY) license (http://creativecommons.org/licenses/by/4.0/).
